# Prevalence, microbiological features, and risk factors for periprosthetic joint infections in oncologic patients following tumor resection and megaprosthetic reconstruction

**DOI:** 10.5194/jbji-10-337-2025

**Published:** 2025-09-08

**Authors:** Andreas G. Tsantes, Dimitrios V. Papadopoulos, Stavros Goumenos, Eleni Petrou, Ioannis G. Trikoupis, Anastasios Roustemis, Alexandra Mpakosi, Petros Ioannou, Christos Koutserimpas, Panayiotis Gavriil, Daniele Piovani, Stefanos Bonovas, Panayiotis J. Papagelopoulos, Athanasios Tsakris, Argirios E. Tsantes

**Affiliations:** 1 Laboratory of Haematology and Blood Bank Unit, “Attiko” Hospital, School of Medicine, National and Kapodistrian University of Athens, Athens, Greece; 2 Microbiology Department, “Saint Savvas” Oncology Hospital, 11522 Athens, Greece; 3 Second Department of Orthopaedics, School of Medicine, National and Kapodistrian University of Athens, Athens, Greece; 4 First Department of Orthopaedics, School of Medicine, National and Kapodistrian University of Athens, Athens, Greece; 5 Charité-Universitätsmedizin Berlin, Corporate Member of Freie Universität Berlin, Humboldt-Universität zu Berlin and Berlin Institute of Health, Center for Musculoskeletal Surgery (CMSC), Berlin, Germany; 6 The Royal Orthopedic Hospital, NHS Foundation Trust, Birmingham, UK; 7 Department of Microbiology, General Hospital of Nikaia “Agios Panteleimon”, 18454 Piraeus, Greece; 8 Department of Internal Medicine & Infectious Diseases, University General Hospital of Heraklion, Heraklion, Greece; 9 Orthopaedics Surgery and Sports Medicine Department, FIFA Medical Center of Excellence, Croix Rousse Hospital, Hospices Civils de Lyon, Lyon North University Hospital, Lyon, France; 10 Department of Biomedical Sciences, Humanitas University, Pieve Emanuele, Milan, Italy; 11 IRCCS Humanitas Research Hospital, Rozzano, Milan, Italy; 12 Department of Microbiology, Medical School, National and Kapodistrian University of Athens, Athens, Greece

## Abstract

Periprosthetic joint infection (PJI) after tumor resection and megaprosthetic reconstruction of bone defects is a common complication. The purpose of this study was to evaluate the prevalence of these infections, assess their microbiological profile, and identify perioperative risk factors for these complications. A single-center retrospective cohort study was conducted including 273 patients, who had undergone musculoskeletal tumor resection and megaprosthetic reconstruction. The medical records of these patients were screened for several parameters, including development of postoperative PJI. All reviewed parameters were compared between patients who developed infections and those who did not. Infection developed in 36 patients, indicating an incidence of 13.2 % (95 % confidence interval (CI): 9.4 %–17.8 %). The most common isolated pathogens in patients with PJI included coagulase-negative staphylococci ( n=20 ; 56 %), followed by *Staphylococcus aureus* ( n=9 ; 25 %). Multivariable logistic regression analysis indicated that development of PJI was associated with diabetes (odds ratio (OR): 7.64; 95 % confidence interval (CI): 1.36–42.7; p=0.020 ), a lower albumin level (OR: 0.10; 95 % CI: 0.02–0.49; p=0.005 ), and a prolonged duration of surgery (OR: 4.30; 95 % CI: 1.08–17.1; p=0.038 ).

Our results indicate that certain parameters such as diabetes, low albumin levels, and prolonged duration of surgery are associated with a higher risk of infection.

## 1 Introduction

Periprosthetic joint infection (PJI) is one of the most feared complications in orthopedic surgery, having a significant impact on the morbidity and mortality of patients undergoing implant-related surgeries. The infection rate following total hip or knee arthroplasties is reported to be 0.5 %–2.5 %, while the estimated economic burden is projected to rise up to USD 1.85 billion annually in the United States by 2030 (Zeng et al., 2023; Weinstein et al., 2023; Yoon et al., 2023; Premkumar et al., 2021). Implant-related infections are unfortunately an even more common problem in oncologic patients following tumor resection and reconstruction of bone defects with specialized prostheses, called megaprostheses. The reported rate of surgical site infections following megaprosthetic reconstruction in oncologic patients ranges from 8 % to 35 % (Shehadeh et al., 2010; Tsantes et al., 2023; Gradl et al., 2014).

There are certain predisposing factors for this higher rate of postoperative infections following extensive procedures compared to elective orthopedic surgeries in otherwise healthy patients (Anatone et al., 2020). The nutritional status of patients is closely associated with the development of PJI (Tsantes et al., 2019). Therefore, since malnutrition is commonly encountered in oncologic patients, this subset of the population is especially prone to the development of postoperative infections. Immunodeficiency due to neoplastic disease or chemotherapy is also another predisposing factor; thus, low-virulence pathogens can induce a severe infection in oncologic patients. Last, radiotherapy constitutes another important risk factor for postoperative infections since it has a negative impact on the wound healing potential, often leading to surgical site infections (Griffin et al., 2015; Zimmerli et al., 2004). Although there is extensive research on prevention strategies, diagnostic modalities, and treatment approaches for PJI following elective joint arthroplasties for degenerative arthritis, there is a lack of information regarding the epidemiology, risk factors, and perioperative parameters associated with PJI in oncologic patients with musculoskeletal tumors.

The aim of this study was to evaluate the prevalence of PJI in oncologic patients with musculoskeletal tumors undergoing tumor resection and megaprosthetic reconstruction of long-bone defects. Moreover, we aimed to assess certain demographics, perioperative parameters, and treatment-related parameters in patients who develop infections and to identify risk factors for these complications.

## 2 Materials and methods

A single-center retrospective cohort study was conducted, including patients who had undergone musculoskeletal tumor resection and megaprosthetic reconstruction of long bones over a 20-year period, from January 2005 to January 2024. The study was conducted in the largest referral center for musculoskeletal tumors nationwide. Inclusion criteria were patients with a minimum follow-up of 1 year. The electronic and manual databases of the hospital were searched in order to identify patients who underwent oncologic resection and megaprosthetic reconstruction of long bones. The medical records of these patients were retrieved and manually screened for several parameters and outcomes, including the development of postoperative infections. Infections were defined based on the International Consensus Meeting (ICM) criteria, involving several parameters such as culture results; synovial fluid findings, including synovial fluid white blood cell count; and serological biomarkers such as C reactive protein (CRP), erythrocyte sedimentation rate (ESR), and D-dimer levels (Bauer et al., 2019).

The following perioperative and treatment-related variables were recorded: length of hospital stay (days), duration of surgery (min), use of soft tissue flaps for wound coverage, length of bone resection, use of bone cement for implant fixation, use of silver-coated implants, status of soft tissue coverage (categorized as good, moderate, or poor), and type of antibiotic prophylaxis. Based on the hospital's protocol, antibiotic prophylaxis is usually discontinued when drains are removed. Preoperatively, antibiotics were initiated within 1 h prior to the incision. Moreover, no specific *Staphylococcus aureus* screening and decontamination protocols were followed.

### 2.1 Statistical analysis

Descriptive statistics were used to summarize demographic characteristics, perioperative parameters, and infection-related data. Continuous variables were presented as means ± standard deviations (SDs) or medians with interquartile ranges (IQRs), depending on the data distribution, while categorical variables were expressed as absolute frequencies and percentages. Normality was assessed using the Shapiro–Wilk test.

Comparisons between patients who developed infections (infection group) and those who did not (control group) were conducted using the Wilcoxon rank-sum (Mann–Whitney) test for continuous variables and the Chi-square test (or the Fisher exact test) for categorical variables. To identify independent factors associated with infection, a multivariable logistic regression analysis was performed, with clinically relevant variables and those significant in univariable analyses included as independent predictors. Statistical analysis was conducted using Stata 15.0 (StataCorp, College Station, TX, USA). A p value < 0.05 was considered to be statistically significant for all tests.

## 3 Results

**Figure 1:**
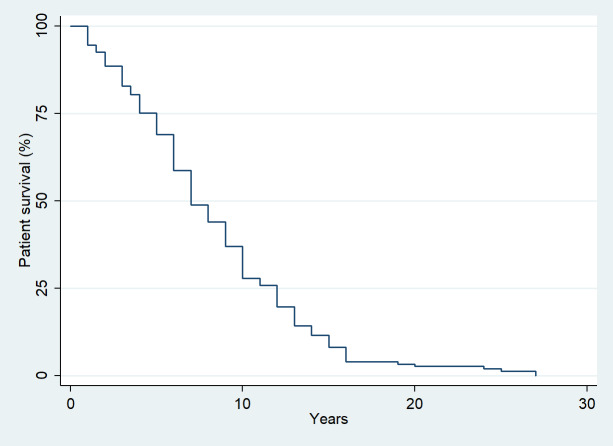
Kaplan–Meier survival plot of the study population.

The median follow-up time was 5 years (interquartile range (IQR): 2–9 years). Overall, 273 oncologic patients who had undergone megaprosthetic reconstruction following tumor resection in long bones were included in the study. Postoperative infection occurred in 36 of them, indicating an infection incidence of 13.2 % (95 % confidence interval (CI): 9.4 %–17.8 %). The median time to infection was 0.5 years (IQR: 0.25–1.1 years) following surgery, while the median survival time in our study population was 7 years (IQR: 5–12 years) (Fig. 1).

### 3.1 Demographics and preoperative parameters

The median age of the study population was 60 years (IQR: 41–70), while patients with or without infection had similar ages (medians: 60 vs. 59 years; p=0.53 ). Gender distribution and smoking status did not differ between patients with and without infection (52.8 % vs. 41.8 % for males, p=0.21 ; 47.2 % vs. 45.1 % for smokers, p=0.83 ). Eight patients (22.2 %) in the infection group and 34 (14.3 %) in the control group had previous surgery at the surgical site ( p=0.22 ). Diabetes was more frequent in the infection group compared to in the control group (61.1 % vs. 15.6 %, p<0.001 ). Moreover, preoperative albumin was lower in the infection group compared to in the control group (medians: 3.0 g dL^−1^ vs. 3.9 g dL^−1^, p<0.001 ), indicating that poor nutritional status was associated with the development of postoperative infection. Demographics and preoperative parameters are summarized in Table 1.

**Table 1:** Demographics of the study population.

	No infection ( n=237 )	Infection ( n=36 )	p value
Age (years)	53.3 ± 20.6; 59 (40–70)	55.4 ± 21.3; 60 (42–71)	0.53
Gender (males, %)	99 (41.8)	19 (52.8)	0.21
Smoking status	107 (45.1)	17 (47.2)	0.83
Diabetes	37 (15.6)	22 (61.1)	<0.001
BMI (kg m^−2^)	27.1 ± 4.4; 27.2 (24.0–30.0)	28.4 ± 5.5; 28.9 (24.7–31.1)	0.21
ASA score			
I II III IV V	75 (31.6) 66 (27.8) 62 (26.2) 32 (13.5) 2 (0.8)	9 (25.0) 9 (25.0) 8 (22.2) 7 (19.4) 3 (8.3)	0.027
Previous surgeries at site	34 (14.3)	8 (22.2)	0.22
Preoperative albumin (g dL^−1^)	3.7 ± 0.6; 3.9 (3.5–4.2)	2.9 ± 0.4; 3.0 (2.6–3.4)	<0.001
Metastatic disease	89 (37.6)	10 (27.8)	0.25

Abbreviation: ASA – American Society of Anesthesiologists and BMI – body mass index. Data are presented with mean ± SD, median (interquartile range), or frequency (percentage) when appropriate.

### 3.2 Location and type of tumors

Regarding the anatomical location of the surgical resection and megaprosthetic reconstruction, the most common location was the proximal femur (44.4 % for infection group vs. 46.8 % for control group, p=0.85 ) followed by the distal femur (33.3 % for infection group vs. 25.7 % for control group, p=0.41 ; Table S1 in the Supplement). Moreover, 5 patients in the infection group (13.9 %) and 12 in the control group (5.1 %) underwent reconstruction of the proximal tibia ( p=0.057 ), while 3 (8.3 %) in the infection group and 49 (20.7 %) in the control group underwent reconstruction of the proximal humerus ( p=0.10 ).

Regarding the type of tumor, the most common primary tumors were osteosarcoma (33.3 % for infection group vs. 20.7 % for control group, p=0.38 ) and chondrosarcoma (16.7 % for infection group vs. 23.2 % for control group, p=0.52 ), followed by and Ewing sarcoma (5.6 % for infection group vs. 8.4 % for control group, p=0.74 ; Table S2). Moreover, 10 patients (27.8 %) in the infection group and 87 (36.7 %) in the control group underwent surgical resection and megaprosthetic reconstruction due to a metastatic tumor ( p=0.35 ), while the frequency of soft tissue sarcoma did not differ between the infection ( n=3 , 8.3 %) and the control groups ( n=9 , 3.8 %; p=0.20 ).

### 3.3 Perioperative and surgical parameters

A longer hospital stay was associated with the development of postoperative infection since the median duration of stay was 13.5 d (IQR: 9.5–23) for patients with infection and 7 d (IQR: 6–10) for patients without infection ( p=0.038 ). Similarly to the hospital stay, the median duration of surgery was longer for patients with infection compared to those without (medians: 195 vs. 150 min; p<0.001 ). Use of soft tissue flaps for wound coverage was more common in patients with infection (16.6 % for infection group vs. 3.8 % control group, p=0.002 ). Moreover, the condition of soft tissue coverage differed between patients with and without infection, with good soft tissue coverage achieved in 86.4 % of patients without infection as opposed to only 37.5 % of patients with infection ( p<0.001 ). The length of the resected bone segment was similar between the two groups (medians: 15 vs. 15 cm; p=0.38 ), while there was also no difference regarding the use of bone cement (58.3 % for infection group vs. 57.8 % for control group, p=0.95 ) and the use of silver-coated implants (41.7 % for infection group vs. 48.5 % for control group, p=0.44 ). A total of 22 patients (61.1 %) in the infection group and 53 (22.4 %) in the control group received preoperative chemotherapy ( p=0.76 ). Also, the proportion of patients who received perioperative radiotherapy did not differ between the two groups (36.1 % for infection group vs. 22.4 % for control group, p=0.07 ). Regarding the type of antibiotic prophylaxis, teicoplanin as a single antibiotic was the most common prophylaxis in the overall population (33.3 % for infection group vs. 42.4 % for control group, p=0.34 ) followed by teicoplanin and tazobactam or piperacillin (27.3 % for infection group vs. 24.2 % for control group, p=0.66 ) and vancomycin (24.2 % for infection group vs. 18.3 % for control group, p=0.47 ; Table 2).

**Table 2:** Perioperative and treatment-related parameters.

	No infection ( n=237 )	Infection ( n=36 )	p value
Hospital stay (days)	10.3 ± 7.5; 7 (6–10)	21.5 ± 19.2; 13.5 (9.5–23)	0.038
Duration of surgery (min)	165.1 ± 53.0; 150 (130–150)	220.2 ± 95.6; 195 (155–250)	<0.001
Use of soft tissue flaps	9 (3.8)	6 (16.6)	0.002
Length of resection (cm)	15.5 ± 4.2; 15 (12–18)	15.9 ± 3.2; 15 (14–18)	0.38
Use of cement	137 (57.8)	21 (58.3)	0.95
Silver-coated implants	115 (48.5)	15 (41.7)	0.44
Soft tissue coverage^a^			
Good Moderate Poor	95/110 (86.4) 13/110 (11.8) 3/110 (2.7)	6/16 (37.5) 7/16 (43.8) 3/16 (18.8)	<0.001 0.004 0.027
Type of antibiotic prophylaxis^b^			
Teicoplanin Teicoplanin and tazobactam or piperacillin Vancomycin and tazobactam or piperacillin Vancomycin Vancomycin and cephalosporin Others	79/186 (42.4) 45/186 (24.2) 12/186 (6.5) 34/186 (18.3) 4/186 (2.2) 12/186 (6.5)	11/33 (33.3) 9/33 (27.3) 3/33 (9.1) 8/33 (24.2) 1/33 (3.0) 1/33 (3.0)	0.34 0.66 0.47 0.47 0.56 0.69

Data are presented with mean ± SD, median and interquartile range (IQR), or frequency (percentage) when appropriate. Abbreviation: BMI – body mass index. ^a^ Data were available for 126 patients. ^b^ Data were available for 219 patients.

### 3.4 Microbiology

In all cases, infection was confirmed with a positive culture. In four patients with diagnosed PJI based on the 2018 ICM criteria for PJI, the culture of synovial fluid culture following joint aspiration was negative. However, all four of these patients underwent surgery for irrigation and debridement, and, intraoperatively, a pathogen was isolated in either synovial fluid culture or tissue culture.

**Figure 2:**
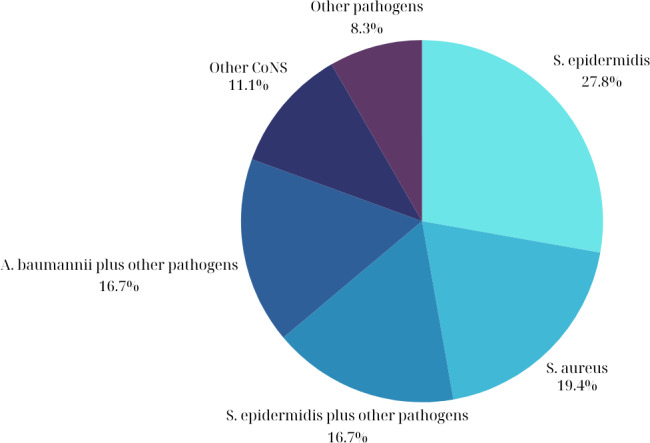
Causative pathogens for periprosthetic joint infections.

The most common isolated pathogens in patients with periprosthetic infections included coagulase-negative staphylococci (CNS) ( n=20 , 56 %) followed by *Staphylococcus aureus* ( n=9 , 25 %) and *Acinetobacter baumannii* ( n=7 , 19 %). Regarding the CNS, methicillin-resistant *Staphylococcus epidermidis* was isolated in 13 patients (36 %), while methicillin-susceptible *Staphylococcus epidermidis* was isolated in 4 patients (11 %). Among the patients with infections due to *Staphylococcus aureus*, methicillin-resistant *Staphylococcus aureus* was isolated in eight patients (22 %), and methicillin-susceptible *Staphylococcus aureus* was isolated in one patient (2.7 %). The most common type of infection was a monomicrobial infection due to methicillin-resistant *Staphylococcus epidermidis* ( n=7 , 19.4 %) followed by a monomicrobial infection due to methicillin-resistant *Staphylococcus aureus* ( n=6 , 16.7 %; Fig. 2). Among the 36 patients with infections, gram-negative pathogens were isolated in 11 (31 %) of them, while fungal infections were present in 6 (17 %). Regarding the six patients with fungal infections, in one of them, the only isolated microorganism was fungus (*Aspergillus* spp.), while, in another patient, two pathogens were isolated, including *Staphylococcus capitis* and *Aspergillus* spp. In the remaining four patients, multiple pathogens such as *S. epidermidis*, *S aureus*, and *A. baumannii* were isolated, with *Candida* spp being one of them. Interestingly, polymicrobial infections ( >1 pathogen) were evident in 14 patients (39 %) (Table S3).

### 3.5 Outcomes

**Figure 3:**
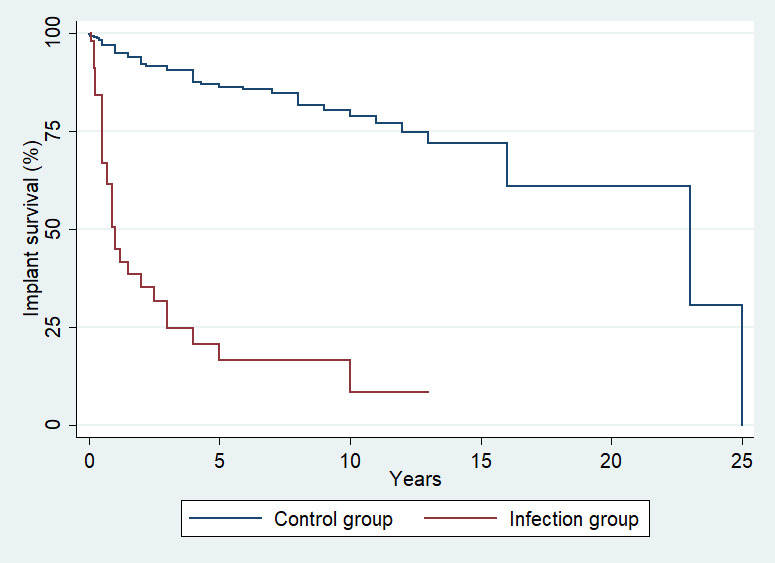
Kaplan–Meier implant survival plots for the infection and control groups.

Among the 36 patients with infection, a second operation was required in 26 (72 %) of them. Regarding the type of reoperation, 14 patients (39 %) underwent irrigation and debridement, 7 (19.4 %) underwent one- or two-stage revision surgery with implant removal and replacement (five patients underwent two-stage revision, and two patients underwent one-stage revision), 3 (8.3 %) had the implants removed and an antibiotic spacer inserted, while 2 (5.5 %) had the limb amputated. The remaining 10 patients who did not undergo a second operation were treated with suppressive antibiotics. The median Musculoskeletal Tumor Society score was similar between the infection and the control groups (21 vs. 23, p=0.10 ). The median implant survival time until revision or removal for patients with infection was 0.6 years (IQR: 0.25–2.2), which is significantly shorter than the median implant survival time for patients without infection (4.0 years; IQR: 2.0–7.0; p<0.001 ) (Fig. 3).

### 3.6 Multivariable analysis

Based on their clinical relevance and the results of univariable regression analyses (significance set at p<0.05 ), the following variables were included in the multivariable model as independent variables: age, gender, body mass index (BMI), diabetes, ASA score, use of soft tissue flaps for wound coverage, preoperative albumin, duration of drain use, condition of soft tissue coverage (good vs. moderate or poor), and duration of surgery (Table 3). The duration of hospital stay was not included in the model since a prolonged hospital stay may reflect a consequence of the infection itself and not a cause for the development of infection. Multivariable logistic regression analysis indicated that development of PJI was associated with diabetes (odds ratio (OR): 13.80; 95 % confidence interval (CI): 2.72–70.02; p=0.002 ), low albumin level (OR: 0.37 per one-unit increase; 95 % CI: 0.15–0.92; p=0.033 ), and a prolonged duration of surgery (OR: 1.01 per one-unit increase; 95 % CI: 1.0003–1.02; p=0.011 ).

**Table 3:** Results of the multivariable logistic regression analysis for development of infection (dependent variable) with age, gender, BMI, diabetes, ASA score, use of flaps, preoperative albumin, condition of soft tissue coverage (good vs. moderate or poor), and duration of surgery, included as independent variables.

	OR	95 % CI	p value
Age (per 1-year increase)	1.01	0.97–1.06	0.44
Gender (male vs. female)	2.34	0.64–8.52	0.26
BMI (per one-unit increase)	0.92	0.78–1.09	0.37
ASA score (per one-unit increase)	0.64	0.27–1.49	0.30
Diabetes (yes vs. no)	13.80	2.72–70.02	0.002
Preoperative albumin (per one-unit increase)	0.37	0.15–0.92	0.033
Soft tissue coverage (good vs. moderate or poor)	0.76	0.19–3.08	0.70
Use of flaps (yes vs. no)	0.18	0.003–1098.5	0.70
Duration of surgery (per 1 min increase)	1.01	1.003–1.02	0.011

Abbreviations: BMI – body mass index, OR – odds ratio, and 95 % CI – 95 % confidence interval.

## 4 Discussion

The detrimental impact of PJI on the prognosis of oncologic patients undergoing surgery is well documented. These infections are associated with a longer hospital stay, while the rate of amputation following the development of these complications ranges from 23.5 % to 87 % (Shehadeh et al., 2010; Jeys et al., 2005). Despite the recent advances in antibiotic prophylaxis, prevention strategies, and the development of implants with enhanced antimicrobial properties, the rate of PJI following megaprosthetic reconstruction in oncologic patients is still high, up to 34 % (Nobile et al., 2015; Haijie et al., 2018). In line with the reported prevalence of these infections in the literature, we found that the infection rate in our study cohort was 13.2 %. Our results supported the presence of a causal association between infections and diabetes, low preoperative albumin levels, and a prolonged duration of surgery.

Multiple studies have shown that patients with diabetes have a higher risk for development of PJI following joint arthroplasties (Jamsen et al., 2012; Duensing et al., 2019; Ahmad et al., 2022). The long-term hyperglycemia associated with diabetes mellitus has a deteriorating impact on the immune system, resulting in decreased leukocyte activity (Kurtz et al., 2008). Moreover, the wound healing potential is impaired in diabetic patients due to micro-angiopathy. The local ischemic environment due to this micro-angiopathy not only decreases the wound healing potential but also has a negative effect on the delivery of antibiotics to the surrounding soft tissues. In a recent meta-analysis including 119 244 patients who underwent total knee arthroplasty, the prevalence of periprosthetic joint infection was 1.9 % in diabetic patients, while, in non-diabetic patients, this rate was 1.2 % (Ahmad et al., 2022). The authors of this study concluded that the risk of developing infection was 1.96 times higher in diabetic patients compared to in non-diabetic patients. In orthopedic oncology, Gradl et al. (2014) conducted a single-center study evaluating risk factors for postoperative surgical site infections in 1304 patients following any oncological surgical procedure. As opposed to our results, Gradl et al. (2014) found that diabetes was not associated with the development of infections. However, this could be attributed to the fact that the authors of this study evaluated any type of oncologic surgical procedure; therefore, less extensive procedures without the use of implants were also included.

The effect of malnutrition on the risk of developing postoperative infections in orthopedic surgery is well documented (Tsantes et al., 2019, 2020). Malnutrition is associated with reduced lymphocytes and impaired immune system functioning; therefore, a dysregulated activity against pathogens is evident in malnourished patients. Moreover, collagen synthesis is reduced in malnourished patient; thus, wound healing problems are more common in these patients (Gherini et al., 1993; Jaberi et al., 2008; Del Savio et al., 1996). In a meta-analysis including > 250 000 patients following total knee and hip arthroplasties, it was shown that the malnourished patients were 3.6 times more likely (OR: 3.62; 95 % CI: 2.33–5.64) to develop periprosthetic joint infections compared to patients with normal nutritional status (Tsantes et al., 2019). In oncology orthopedic surgery, there is only one study by Meijer et al. (2017) evaluating the nutritional status of patients undergoing reconstructive shoulder surgery for proximal humerus tumors. In this study, including 150 oncologic patients, the authors also found that lower albumin levels were independently associated with postoperative infections. Our study is the first one evaluating the nutritional status of patients undergoing reconstructive surgery for malignant tumors in any long bone, which is in line with the results of Meijer at al. (2017).

The association between a prolonged duration of surgery and higher risk of infections stands on plausible biological grounds, with several studies demonstrating a positive association between longer duration of surgery and higher risk of infection in elective joint arthroplasties (Pugely et al., 2015; Bozic et al., 2014; Zhu et al., 2015; Namba et al., 2013). With prolonged operative time, the exposure of open incisions to the environment is prolonged; therefore, the risk of pathogen contamination is increased. Moreover, prolonged duration of surgery is associated with tissue desiccation, which also predisposes the patient to contamination, while tissue concentration of antibiotics is decreased. Cheng et al. (2017) conducted a comprehensive review and found that the risk for postoperative infection was doubled if the duration of surgery was > 1–4 h and almost tripled if the duration was > 5 h. In orthopedic oncology, De Gori et al. (2017) also conducted a systematic review including 2510 patients with musculoskeletal tumors and showed that a prolonged duration of surgery ( > 2.5 h) was found to be associated with a higher risk of infection. In another study including 1521 musculoskeletal oncological procedures with or without implants, the authors found that the duration of surgery was independently associated with the risk of infection (OR: 1.16; 95 % CI: 1.07–1.25) (Gradl et al., 2014).

There are only a few studies evaluating the microbiology of PJI in oncologic patients, indicating that similar pathogens cause PJI in elective arthroplasties and oncological reconstructions. Oncologic patients are immunosuppressed; thus, low-virulence pathogens such as CNS can commonly induce a PJI in these patients. Jeys et al. (2005) evaluated the microbiology of PJI in 1264 oncologic patients after prosthetic reconstruction and found that CNS constituted the most common isolated pathogen in almost half of the patients (48 %), similarly to our study, in which the CNS pathogen was isolated in 56 % of patients. Moreover, the percentage of fungal infections (16 %, 6 out of the 36 patients with PJI) that was revealed in our study is higher compared to the rate of fungal PJI infections in elective non-oncological arthroplasties. This could be explained by the nature of these surgical cases and the overall status of patients since, as opposed to those patients who undergo elective total knee or hip arthroplasties, cancer patients are immunocompromised patients who are susceptible to infections from such microorganisms. Another worrisome finding in our study that has not been reported in other studies is that *Acinetobacter baumannii*, an extremely resistant pathogen with severe consequences, was isolated in 19.4 % of our patients with PJI.

There are certain limitations of this study that should be addressed. The number of patients is relatively small, while studies with larger populations have been also conducted, evaluating risk factors for development of infections following bone tumor resection and megaprosthetic reconstruction. The small population of this study may be the reason that no significant association was found between certain important risk factors for infections such as the length of resection, the tumor location, and the quality of soft tissue coverage. However, this is the largest study evaluating a comprehensive set of perioperative and treatment-related parameters, such as preoperative albumin levels in patients with long-bone tumors. Moreover, our results rely on the quality of data retrieved from electronic and manual medical records, which could potentially have contained errors or omissions.

## 5 Conclusions

Despite the recent advances in prevention strategies and the development of newer implants with antimicrobial properties, the risk of periprosthetic infections following musculoskeletal tumor resection and megaprosthetic reconstruction is high. Our results indicate that certain parameters such as diabetes, low albumin levels, and a prolonged duration of surgery are independently associated with a higher risk of infection. Therefore, a multidisciplinary approach is needed to mitigate the risk of developing these detrimental complications by applying certain preventive measures. Strategies targeted towards glycemic control and nutritional status optimization are of great significance, while parameters that can affect the operating time – such as through pre-operative planning, surgeon and operating staff experience, and access to equipment – can also have a positive impact on PJI prevention. Future studies evaluating whether optimizing these modifiable risk factors would lead to a decreased rate of postoperative infections in oncologic patients would be valuable.

## Supplement

10.5194/jbji-10-337-2025-supplement The supplement related to this article is available online at https://doi.org/10.5194/jbji-10-337-2025-supplement.

## Data Availability

The datasets used and/or analyzed during the current study are available from the corresponding author on reasonable request.

## Appendix

The supplement related to this article is available online at https://doi.org/10.5194/jbji-10-337-2025-supplement.
