# Supplementary material for: Prevalence, microbiological features, and risk factors for periprosthetic joint infections in oncologic patients following tumor resection and megaprosthetic reconstruction
Source: J Bone Jt Infect. 2025 Sep 8;10(5):337–45. doi: 10.5194/jbji-10-337-2025 (PMC12590581; doi:10.5194/jbji-10-337-2025)
Supplement: The supplement related to this article is available online at https://doi.org/10.5194/jbji-10-337-2025-supplement. [file jbji-10-337-2025-supplement.pdf]

Supplement of J. Bone Joint Infect., 10, 337–345, 2025
https://doi.org/10.5194/jbji-10-337-2025-supplement
© Author(s) 2025. CC BY 4.0 License.

JBJI
Journal of Bone and Joint Infection
Open Access

Supplement of

Prevalence, microbiological features, and risk factors for periprosthetic joint infections in oncologic patients following tumor resection and megaprosthetic reconstruction

Andreas G. Tsantes et al.

Correspondence to: Andreas G. Tsantes (andreas.tsantes@yahoo.com)

The copyright of individual parts of the supplement might differ from the article licence.

Table S1. Location of the tumors.

Location of endoprosthestic reconstruction	No infection (n=237)	Infection (n=36)	p-value
Proximal femur	111 (46.8)	16 (44.4)	0.85
Distal femur	61 (25.7)	12 (33.3)	0.41
Knee	4 (1.7)	0 (0.0)	0.99
Proximal tibia	12 (5.1)	5 (13.9)	0.057
Proximal humerus	49 (20.7)	3 (8.3)	0.10

Data are presented as frequency (percentage).

Table S2. Type of tumors.

Tumor	No infection (n=237)	Infection (n=36)	p-value
Osteosarcoma	49 (20.7)	12 (33.3)	0.38
Chondrosarcoma	55 (23.2)	6 (16.7)	0.52
Ewing	20 (8.4)	2 (5.6)	0.74
Giant cell tumor	6 (2.5)	1 (2.8)	0.99
Other	11 (4.6)	2 (5.6)	0.68
Metastatic tumor	87 (36.7)	10 (27.8)	0.35
Soft tissue sarcoma	9 (3.8)	3 (8.3)	0.20

Data are presented as frequency values (percentage).

Table S3. Microbiology of periprosthetic joint infections.

Pathogens	Patients (n=36)
Methicillin-Resistant Staphylococcus epidermidis	7 (19.4%)
Methicillin-Sensitive Staphylococcus epidermidis	3 (8.3%)
Staphylococcus hominis	1 (2.8%)
Staphylococcus capitis	1 (2.8%)
Methicillin-Resistant Staphylococcus aureus	6 (16.7%)
Methicillin-Sensitive Staphylococcus aureus	1 (2.8%)
Methicillin-Resistant Staphylococcus epidermidis	
+ Pseudomonas aeruginosa, Acinetobacter baumannii, Candida spp.	1 (2.8%)
+ Methicillin-Resistant Staphylococcus aureus	1 (2.8%)
+ Enterobacter clocae	1 (2.8%)
+ Klebsiella pneumoniae	1 (2.8%)
+ Klebsiella pneumoniae, Pseudomonas aeruginosa	1 (2.8%)
+ Candida spp., Methicillin-Resistant Staphylococcus aureus	1 (2.8%)
Methicillin-Sensitive Staphylococcus epidermidis + Candida spp.	1 (2.8%)
Staphylococcus capitis + Aspergillus spp.	1 (2.8%)
Acinetobacter baumannii	
+ Enterococcus faecium	1 (2.8%)
+ Proteus mirabilis	2 (5.6%)
+ Klebsiella pneumoniae	1 (2.8%)
+ Pseudomonas aeruginosa	1 (2.8%)
+ Enterococcus faecalis, Klebsiella Pneumoniae, Candida spp,	1 (2.8%)
Aspergillus spp.	1 (2.8%)
Streptococcus oralis	1 (2.8%)
Achromobacter spp.	1 (2.8%)
Infections including Gram negative pathogens	11 (30.6%)
Fungal infections	6 (16.7%)
Polymicrobial infections	14 (38.9%)

Data are presented with frequency (percentage).
